# Examination of the Relationship Between Pain Intensity, Pain Perceptions, and Kinesiophobia in Patients with Non-Specific Chronic Musculoskeletal Pain

**DOI:** 10.3390/muscles4030027

**Published:** 2025-08-04

**Authors:** Sofia Sgourda, Maria Loulla, Eirini Zisiopoulou, Krystalia Katsiou, Sofia Nikolaidi, Ioannis Kyrosis, Anna Christakou

**Affiliations:** 1 Department of Physiotherapy, School of Health Sciences, University of Peloponnese, 23100 Sparta, Greece; sofiasgourda@gmail.com (S.S.); mariadouloulla19@gmail.com (M.L.); zisio.eirini@gmail.com (E.Z.); kristaliakats@gmail.com (K.K.); sofianikol04@gmail.com (S.N.); johnkyr19@gmail.com (I.K.); 2 Laboratory of Biomechanics, Department of Physiotherapy, School of Health Sciences, University of Peloponnese, 23100 Sparta, Greece

**Keywords:** chronic musculoskeletal pain, kinesiophobia, pain perceptions

## Abstract

Chronic musculoskeletal pain negatively affects patients’ quality of life, and pain perceptions may significantly influence rehabilitation outcomes. This study investigated the relationships among pain intensity, pain perceptions, and kinesiophobia in individuals with chronic musculoskeletal pain. No previous studies have examined these variables in combination. A cross-sectional observational study was conducted with 37 participants with non-specific chronic musculoskeletal pain for at least 6 months, affecting the neck (n = 8), lower back (n = 18), upper limbs (n = 5), lower limbs (n = 5), or shoulder (n = 1). The following validated tools were used: (a) Pain Beliefs and Perceptions Inventory (PBPI), (b) the Tampa Scale for Kinesiophobia (TSK), and (c) the Short-Form McGill Pain Questionnaire (SF-MPQ). Spearman r correlation analyses were performed. Total kinesiophobia scores were positively correlated with (a) total pain intensity (McGill score) (r = 0.37, *p* = 0.022), (b) present pain intensity (PPI) (r = 0.52, *p* = 0.001), (c) pain duration (r = 0.51, *p* = 0.001), (d) the “mystery” factor of pain perception (r = 0.41, *p* = 0.013), and (e) the Visual Analogue Scale (VAS) (r = 0.42, *p* = 0.009). The total pain perception scores were positively associated with the “fear of injury” factor of kinesiophobia (r = 0.36, *p* = 0.028). The McGill pain scores were strongly correlated with both PPI (r = 0.63, *p* = 0.001) and VAS (r = 0.51, *p* = 0.001). There is a significant relationship between pain perception and kinesiophobia levels in patients with chronic musculoskeletal pain. Limitations of the study include a small and heterogeneous sample regarding pain localization. Further research is required using larger, more homogeneous populations to confirm the present findings.

## 1. Introduction

Chronic musculoskeletal pain is a prevalent and distressing perceptual experience affecting a great number of patients worldwide. The management of chronic pain, particularly through physiotherapy, poses considerable challenges due to its multifactorial nature. Contemporary therapeutic frameworks emphasize the biopsychosocial model, which requires physiotherapists not only to address the biological components of pain but also to assess and intervene on psychological and social factors that contribute to the pain experience [1]. A critical aspect of this model involves understanding patients’ beliefs and perceptions about pain, which can significantly influence treatment outcomes. Pain perception is a subjective experience affected by sensory, emotional, cognitive, and behavioral dimensions [2]. There is not a single “pain center” in the body. In reality, the peripheral nervous system activated by pain stimuli and various brain regions are responsible for the processing of pain [3].

Kinesiophobia, defined as an excessive and irrational fear of physical movement due to a feeling of vulnerability to injury or re-injury [4], acts as a substantial barrier to effective rehabilitation [5]. It contributes to the reduction of physical function [4]. It promotes pain-avoidant behavior and maladaptive motor strategies, ultimately exacerbating disability and limiting patient autonomy in pain self-management [6,7]. An increase in fear avoidance levels of athletes at injury onset could delay the return to competition [8]. A longitudinal study found that kinesiophobia could predict physical activity levels more efficiently than pain characteristics among older people with chronic musculoskeletal pain [9]. Kinesiophobia is not just involved in musculoskeletal problems but also in other pathological situations. For example, it can negatively affect the physical activity level of patients with hemophilia and lead to hemarthrosis [10]. Fear of injury, which is a somatic focus of kinesiophobia, implies that pain is a result of tissue damage and is characterized by hypervigilance towards bodily sensations. It can lead to a reduction of physical activity and may also be linked to health-related quality of life and pain catastrophizing [11].

Musculoskeletal pain is common to most people at some point in their life [12]. Pain persists in the absence of structural or traumatic pathology, with psychosocial variables playing a pivotal role in pain chronicity. Kinesiophobia, in this context, not only intensifies the subjective pain experience but also undermines engagement in therapeutic exercises and activities essential for recovery. Despite the explosion of scientific research studying the role of kinesiophobia in pain conditions, the majority have engaged with musculoskeletal system problems, especially with low back and neck pain [13,14,15,16].

Current literature indicates a positive association between kinesiophobia and pain intensity in individuals with chronic musculoskeletal pain. However, the strength of this association can vary depending on the population studied and the assessment methods employed. A systematic review by Luque-Suarez et al. concluded that elevated levels of kinesiophobia are associated with increased pain intensity and reduced quality of life in individuals with chronic musculoskeletal pain [7]. Nonetheless, some studies have found weaker or non-significant associations, suggesting that other factors, such as psychological state, pain duration, and coping strategies, may influence this relationship. Therefore, while a general positive correlation exists, the strength and clinical significance of the association between kinesiophobia and pain may vary.

This study differs substantially from previous research in the field in terms of the types of factors selected for investigation. Unlike other studies that focus on a limited range of variables, the present study adopted a broader research framework, incorporating multiple factors with potential synergistic effects. The present study investigated the interrelationship between pain intensity, pain perception, and kinesiophobia in individuals suffering from chronic non-traumatic musculoskeletal pain. This study emphasizes the clinical significance of patients’ pain-related beliefs and psychological responses, offering physiotherapists deeper insights to facilitate more targeted and effective interventions. The study hypothesis posited that pain intensity, pain perceptions, and kinesiophobia would demonstrate a direct positive association, while kinesiophobia would be positively correlated with pain duration.

## 2. Materials and Methods

### 2.1. Design

This was a cross-sectional observational study. This study has been registered and approved by the Ethics Committee of the School of Health Studies of the University of Peloponnese (number: 816-14-1-2025).

### 2.2. Sample

Thirty-seven patients with non-specific chronic pain participated in the study. A priori power analysis was conducted using G*Power version 3.1.9.7, which showed that at least a sample of 29 participants is necessary in a correlation: bivariate normal model with an 80% power for detecting a large effect and a significance criterion of α = 0.05.

The sample’s inclusion criteria were

(a) Being diagnosed with non-specific chronic pain (lasting more than three months) [17].
(b) Aged between 18 and 70 years, including both sexes.
(c) Have undergone physiotherapy, with a maximum of 1–2 sessions.

The exclusion criteria for patient selection in this study were

(a) History of cancer.
(b) Pregnancy during patient selection.
(c) Presence of any mental disorder.
(d) Physiotherapy intervention in the last 12 months.

### 2.3. Instruments

*(a)*  *Tampa Scale for Kinesiophobia (TSK), (Miller et al., 1991, as reported in [18])*

While TSK was designed in 1991 for low back pain, it was not published until 1995 [19]. TSK is one of the most well-known self-report measures evaluating fear related to pain. This 5-point Likert scale accepts answers from 0 (strongly agree) to 4 (strongly disagree). The original instrument consists of 17 items. The total score ranges from 17 to 68 points, with a TSK score ≥ 37 points indicating kinesiophobia. This questionnaire has high internal consistency (Cronbach’s α, 0.79) and excellent test-retest reliability (intraclass correlation coefficient, 0.90) [20]. Among the shortened versions of TSK, TSK-11 is the most widely used. The various models of TSK comprise one, two, or three factors [21]. It is generally admitted that somatic focus (fear of injury) and activity avoidance are the 2 factors of TSK. The Greek version of TSK presents satisfactory internal consistency (Cronbach’s α, 0.74) and test-retest reliability (intraclass correlation coefficient, 0.78) [21].

*(b)*  *Pain Beliefs and Perceptions Inventory (PBPI) [22]*

This questionnaire evaluates the pain perceptions and beliefs in patients with chronic pain. It takes little time to complete; it is user-friendly and has excellent psychometric properties. The initial version of PBPI consisted of 16 items with 3 subscales: time, mystery, and self-blame. The time factor was later divided into ‘acceptance’ and ‘constancy’. This questionnaire has been adapted for the Greek population with chronic pain. The Greek version is internally consistent (α = 0.89–0.96) and has good stability (intraclass correlation coefficients = 0.73–0.82) [23].

*(c)*  *Short-Form McGill Pain Questionnaire (SF-MPQ), [24]*

SF-MPQ is a useful tool for testing the quality of pain in situations that the standard questionnaire cannot. The main component of the SF-MPQ consists of 15 descriptors (11 sensory, 4 affective) rated on an intensity scale as 0 = none, 1 = mild, 2 = moderate, or 3 = severe. This questionnaire also includes the Present Pain Intensity (PPI) index of the standard MPQ and a Visual Analogue Scale (VAS). The correlations between the short and standard forms of the MPQ are significantly high, and it only takes 2–5 min to administer. The Greek version of the SF-MPQ has satisfactory internal consistency (Cronbach’s α = 0.71) and a significant association between the affective and sensory components of the questionnaire (r = 0.55) [25].

### 2.4. Procedure

Authors contacted the participants, and they informed them about (a) the aim of the study, (b) their voluntary involvement, and (c) the protection of their privacy data. Volunteers after their registration to the study (convenience sample) and the evaluation regarding the inclusion criteria were asked to sign an official informed consent paper and fill in the questionnaires. All the questionnaires were self-administered. Also, all questionnaires were distributed to participants immediately after their physiotherapy sessions. This approach ensured consistency in timing and minimized external influences on the responses. The distribution took place in a quiet and calm environment, free from noise and distractions, allowing participants to focus and respond without pressure or interruptions.

### 2.5. Statistical Analyses

Descriptive statistics (means, standard deviations, skewness, and kurtosis) measures were used to organize the demographic dataset of the sample and their answers to the questionnaires. Also, Kolmogorov–Smirnov tests were performed to assess the normality of the variables. Non-parametric analyses, including Spearman’s rank correlation coefficient (Spearman r) with effect sizes, were used to examine the relationships between questionnaires’ variables. The effect sizes were interpreted as small, r = 0.1; medium, r = 0.3; and large, r = 0.5 [26]. We also applied Bonferroni correction to multiple Spearman correlation analysis (thus changing alpha to 0.05/3 = 0.016) in order to control the probability of making at least one type I error across the multiple correlations. The statistical package SPSS 28.00 was used.

## 3. Results

The sample consisted of 37 patients of the white/Caucasian race (from Europe/Greece) (24 women, 13 men) with a mean age of 48 years (SD = 15.12). The majority reported chronic pain in the lumbar region (48.6%), followed by the cervical spine (21.6%) and upper and lower limbs (27%). The most common cause of pain was onset after activity (62.2%), followed by falls (18.9%) and suddenly induced pain (18.9%). Also, the mean duration of pain was 2.86 years (SD = 1.81). Other demographic variables included participants’ education level and compensation from the employer (Table 1). The demographic characteristics of the participants, as well as the variables of the questionnaires, are presented in Table 1.

The results revealed that for *p* < 0.05, the total score of the Tampa Scale for Kinesiophobia (TSK) demonstrated a significant positive correlation with the total score of the Short-Form McGill Pain Questionnaire (SF-MPQ) (r = 0.37, *p* = 0.022, medium effect size). Also, the total score of the Pain Beliefs and Perceptions Inventory (PBPI) was positively correlated with the “fear of injury” subscale of the TSK (r = 0.36, *p* = 0.028, medium effect size) (Table 2).

**Figure 1:**
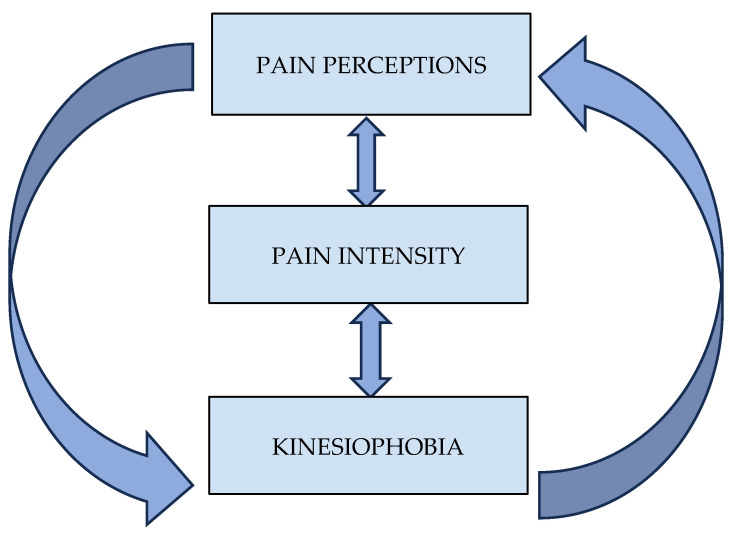
Interconnections between pain perceptions, pain intensity, and kinesiophobia.

Additionally, for *p* < 0.016 (Bonferroni correction), the total score of the TSK was positively correlated with (a) the Present Pain Intensity (PPI) index (r = 0.52, *p* = 0.001, large effect size), (b) the pain duration (r = 0.51, *p* = 0.001, large effect size), (c) the “Mystery” subscale of the PBPI (r = 0.41, *p* = 0.013, medium effect size), and (d) the Visual Analogue Scale (VAS) (r = 0.42, *p* = 0.009, medium effect size). Finally, the total score of the SF-MPQ was significantly correlated with both the PPI (r = 0.63, *p* = 0.001, large effect size) and the VAS score (r = 0.51, *p* = 0.001, large effect size). Further correlations between the questionnaire’s variables are shown in Table 2. A visual summary of the study variables’ interconnection can be seen in Figure 1.

## 4. Discussion

The present study investigated the associations between kinesiophobia, pain perception, and pain intensity in individuals experiencing non-specific chronic musculoskeletal pain. Τhe findings, aligned with our study hypothesis, reveal statistically significant positive correlations among these variables, supporting and extending the existing literature on the psychosocial mechanisms underlying chronic pain. Specifically, the study identified a robust correlation between kinesiophobia and pain intensity, as well as an association with pain perception, particularly the ‘mystery’ factor, which captures uncertainty or lack of clarity about the pain experience.

Τhe observed relationships between pain intensity, pain perception, and kinesiophobia in patients with non-specific chronic musculoskeletal pain can be effectively explained through several well-established theoretical frameworks. The Fear-Avoidance Model of Pain posits that when individuals interpret pain as threatening, they may develop a fear of movement (kinesiophobia), which leads to avoidance behaviors, ultimately reinforcing disability and sustaining chronic pain [27]. This aligns with the study’s findings of significant positive correlations between kinesiophobia, pain intensity, and the duration of symptoms. Complementing this, the Cognitive-Behavioral Theory of Pain emphasizes the role of dysfunctional beliefs and maladaptive thought patterns—such as catastrophizing or perceiving pain as mysterious or uncontrollable—which can exacerbate pain perception and hinder recovery [28] (pp. 132–154). These cognitive constructs are evident in the associations observed between pain beliefs and kinesiophobic avoidance behaviors.

Our results are consistent with neurophysiological studies investigating the nature of pain. According to Stegemann et al. [29], a pain experience encodes a fear memory stored in specific prefrontal cortex neurons. After a novel pain situation later in life, these neurons are reactivated, intensifying the pain perception and driving pain chronicity. Several other brain regions of the fear network are also activated during a pain experience (amygdala, anterior cingulate cortex, and hippocampus) [30]. Treatment interventions deleting long-term fear memory are critical. These interventions may target the prefrontal mechanisms for enhancing the results. Moreover, mesolimbic and prefrontal areas involved in the regulation of emotional reactions communicate with regions of the brainstem and sensorimotor centers affecting descending pain modulation [31,32].

Luque-Suarez et al. [33] investigated chronic shoulder pain and reported that kinesiophobia was significantly associated with both pain intensity and disability, underlining the behavioral implications of pain-related fear in musculoskeletal conditions. These results are in alignment with the present study, which confirms a strong relationship between pain levels and elevated kinesiophobia scores. However, while Luque-Suarez et al. [33] primarily emphasized functional outcomes and clinical disability, our study introduces pain perception—especially the element of cognitive ambiguity—as a critical intermediary factor. This offers a broader psychosocial framework that goes beyond physical impairment and incorporates how patients cognitively process and interpret their pain experiences.

It is worth noting that some studies have found opposite results regarding kinesiophobia and pain intensity levels. Markfelder and Pauli [34] reported, in their meta-analysis of 253 studies, a small to moderate positive correlation between fear of pain and pain intensity. Specific demographics such as age, location of pain, first-time pain episode, and sensitivity to anxiety were recognized as factors influencing the above association. Other studies [35,36] show association magnitudes similar to Markfelder and Pauli [34] between fear of pain and intensity. In a systematic review, 13 out of 38 studies with cross-sectional analyses found no significant correlation between those variables [7]. Fear related to pain has been described so far with a bunch of operational definitions. Pain-related fear, fear avoidance beliefs, fear of movement, and kinesiophobia are some examples. This inconsistency of terms usually leads to confusion and difficulties in the interpretation and comparison of the results.

In the study of Georgoudis, Raptis, and Koutserimpas [21], there was an enhanced association between the Greek version of the Tampa Scale for Kinesiophobia (TSK) and the Visual Analogue Scale (VAS), compared to the literature examining this relationship [7,37,38,39,40]. In our study, a low correlation was observed between the Greek version of the TSK and the VAS scale, but it was still greater than the findings of the above literature.

Cleland et al. [41] examined the psychometric properties of fear-avoidance assessment tools (Fear-Avoidance Beliefs Questionnaire and Tampa Scale for Kinesiophobia) in patients with neck pain, emphasizing the importance of valid and reliable measures for understanding pain-related fear. The current study extends this work by applying the TSK alongside tools that capture subjective pain perception, such as the Pain Beliefs and Perceptions Inventory (PBPI). This integrative approach enables the identification of specific cognitive components, such as the ‘mystery’ factor, which was found to be significantly correlated with kinesiophobia. This suggests that uncertainty or lack of explanatory frameworks about the pain experience may lead to reinforcement of avoidance behaviors. In general, the mysteriousness of pain could be a result of the controversial nature of a disease and makes it difficult for patients to describe their experience. “Medically unexplained” pain and the unpredictability of a specific condition could be irritating. Encouraging patients to seek solutions rather than explanations may play an important role in relieving their symptoms [42].

Larsson et al. [43] evaluated the role of kinesiophobia in older adults with chronic pain and found significant associations with both pain intensity and emotional/cognitive factors such as anxiety and catastrophizing. The present findings extend these results by confirming the role of pain intensity and introducing pain perception as a distinct cognitive variable. While Larsson et al. [43] focused on affective aspects (e.g., anxiety), this study adopts a more cognitive-evaluative angle by examining how individuals interpret or fail to interpret their pain.

From a clinical perspective, the results emphasize the need to address not only pain intensity but also patients’ cognitive representations and beliefs about their pain. In the study by Thomas et al. [44], the evaluation of kinesiophobia is highlighted as a critical component in the rehabilitation of patients with chronic low back pain. The authors emphasize that kinesiophobia is strongly associated with the level of functional disability and the overall quality of life in these patients. Interestingly, the study found that kinesiophobia was not directly correlated with pain intensity. Instead, it was significantly linked to the degree of activity limitation experienced by patients. This suggests that fear-related avoidance behaviors may play a more substantial role in long-term disability than pain itself.

Additionally, the relationship between pain duration and kinesiophobia supports our hypothesis and the idea that prolonged uncertainty about pain may have a compounding effect on movement-related fear. In a study comparing the effectiveness of exercise on pain in patients with high and low kinesiophobia levels, Vaegter et al. [45] found increased pain intensity as the level of kinesiophobia increased in this group compared with the low-level-of-kinesiophobia group, but the pain duration was not different in these two groups.

The systematic assessment of kinesiophobia as part of the initial clinical evaluation should be adopted by rehabilitation personnel. Incorporating validated measurement tools, such as the TSK, may facilitate more targeted and effective rehabilitation strategies. Recognizing and addressing kinesiophobia early in the treatment process may be essential for improving patient outcomes and promoting long-term functional recovery [7]. Psychoeducational interventions that reduce uncertainty and clarify pain mechanisms may be crucial in disrupting fear-avoidance cycles and improving functional οutcomes. Ho et al. [46] underscore the relevance of psychological interventions, including psychoeducation, in addressing psychosocial contributors to chronic low back pain. They concluded that interventions incorporating psychoeducational components are effective in reducing maladaptive fear-related cognitions and behaviors, including kinesiophobia and fear-avoidance beliefs. These findings align with the broader evidence base suggesting that targeting patients’ beliefs and understanding of pain through educational frameworks can facilitate functional improvements by mitigating fear-related avoidance patterns. The authors recommend that psychoeducational strategies should be embedded within multimodal treatment programs to optimize outcomes in this patient population.

The total score of the Short-Form McGill Pain Questionnaire (SF-MPQ) in our study was significantly correlated with both the Present Pain Intensity (PPI) and the VAS score, confirming internal consistency across subjective pain intensity measurements. Georgoudis, Watson, and Oldham [25] showed that the affective score correlates with the VAS in contrast to the sensory one. They further explained this fact, supporting that patients may have a tendency to complete VAS according to the emotional state of their pain experience. PPI and the SF-MPQ were not associated in their study. This possibly indicates that unidimensional scales are not adequately able to describe the pain experience as completely as the rating scales with multiple dimensions. Most research should be conducted evaluating the relationship between SF-MPQ, PPI index, and VAS.

A strength of this study is its multidimensional methodology, combining validated tools (TSK, VAS, SF-MPQ, PBPI) to assess physical, affective, and cognitive features of chronic pain. The distinctive contribution of the current study lies in highlighting ‘mystery’—a form of cognitive uncertainty—as a key element in the fear-avoidance model. This may explain why individuals with similar pain intensities exhibit different levels of kinesiophobia, depending on how they understand and appraise their pain.

The small sample size is one of the limitations that restricts the statistical power of the findings and limits the generalizability of the results to broader populations. Furthermore, the heterogeneity in the anatomical location of pain among participants—ranging from the cervical and lumbar spine to upper and lower extremities—may have introduced variability that affects the interpretation of the observed associations between pain perception; pain intensity; and kinesiophobia.

Future studies should replicate these findings in larger, more homogenous populations regarding anatomical area and in various types (specific etiology—neurologic pain) or phases of pain (acute-subacute). Moreover, future studies could include different anthropometric and demographic characteristics (Body Mass Index, age, etc.) to investigate how these features affect the interrelationship between kinesiophobia and pain intensity. Forming a consensus about the concept of kinesiophobia and the application of TSK seems also highly important in the research methodology. Furthermore, in future studies, it is necessary to stratify by age and gender the heterogeneous sample and to investigate if any anatomical location with pain (i.e., neck, shoulder, back, legs, etc.) has worse outcomes in pain beliefs, kinesiophobia, or quality of life. Also, longitudinal or interventional research designs would be more appropriate to test the directionality of the observed associations between variables such as pain, kinesiophobia, anxiety, strength, range of motion, etc. Exploring pain perception and kinesiophobia in response to cognitive-based interventions through longitudinal study designs could lead to more effective therapeutic plans. Moreover, the inclusion of a control group in further research could enhance the clinical relevance of the findings. Finally, future studies applying a regression analysis with a larger sample size could assess the predictive ability of various variables, such as quality of life, kinesiophobia, range of motion to the appearance and severity of chronic musculoskeletal problems in neck, low back, etc.

## 5. Conclusions

The findings of the current study demonstrate that pain intensity, cognitive pain perception—especially its perceived ambiguity—and kinesiophobia are significantly interrelated in individuals with chronic non-specific musculoskeletal pain. Specifically, elevated levels of kinesiophobia were linked to increased pain intensity, longer pain duration, and more maladaptive pain beliefs—particularly the perception of pain as mysterious and the fear-avoidance component. This suggests that patients’ cognitive and emotional interpretations of pain may play a crucial role in their physical function and response to rehabilitation. As far as we know, this is the first study to examine the combined relationship between these three variables. However, the small sample size and the heterogeneity in pain localization across the body represent limitations that may affect the generalizability of the findings. Future research involving larger samples with homogeneity in their characteristics is warranted to further validate and expand upon these results, potentially guiding more targeted physiotherapeutic interventions.

## Figures and Tables

**Table 1:** Demographic characteristics of the participants and questionnaires’ variables.

Characteristics	Minimum Value	Maximum Value	Mean ± SD	Skewness	Kurtosis
Age (years)	24	70	48 (15.12)	−0.03	−1.24
Pain duration (years)	1	6	2.86 (1.80)	0.27	−1.54
PBPI (total score)	1.84	3.63	2.49 (0.29)	1.68	5.99
Constancy	1.75	3.50	2.54 (0.08)	0.24	−0.58
Acceptance	1.40	3.50	2.59 (0.38)	−0.17	1.91
Self-blame	1	4	2.51 (0.89)	0.04	−1.15
Mystery	1.50	4.50	2.38 (0.65)	0.83	1.62
TSK (total score)	1.35	3.23	2.39 (0.36)	−0.17	1.07
Fear of Injury	1.33	3.33	2.57 (0.36)	−0.74	3.02
Activity Avoidance	1.38	3.25	2.22 (0.09)	0.47	−0.92
SF-MPQ (total score)	1.13	3.33	2.11 (0.67)	0.46	−1.11
PPI	1	5	2.56 (1.03)	0.51	0.2
**Characteristics**		**Answers**		**Frequency (Percentage)**	
Sex		Female Male		24 (64.9) 13 (35.1)	
Family status		Having children No children		20 (54.1) 17 (45.9)	
Educational level		Lyceum University		20 (54.1) 17 (45.9)	
Occupation		Manual labor Intellectual work Retired		20 (54.1) 12 (32.4) 5 (13.5)	
Anatomical site of chronic pain		Neck Lower back Shoulder Upper limbs Lower limbs		8 (21.6) 18 (48.6) 1 (2.7) 5 (13.5) 5 (13.5)	
Cause of pain		During activity Fall Sudden pain		23 (62.2) 7 (18.9) 7 (18.9)	
Visit the doctor		Yes No		37 (100) 0 (0)	
Medicine		No Yes		15 (40.5) 22 (59.5)	
Compensation of the employer		Yes No		37 (100) 0 (0)	

PBPI, Pain Beliefs and Perceptions Inventory; TSK, Tampa Scale for Kinesiophobia; SF-MPQ, Short-Form McGill Pain Questionnaire; PPI, Present Pain Intensity.

**Table 2:** Correlations between questionnaires’ factors.

Variables	PBPI	TC	A	S	M	TSK	Act.Av.	FoI	SFMPQ	PPI	VAS	PD
**PBPI**	1											
**TC**	0.48 **	1										
**A**	0.46 **	0.17	1									
**S**	0.31	−0.32	−0.13	1								
**M**	0.52 **	0.57 **	0.44 **	−0.35	1							
**TSK**	0.26	0.43 **	0.08	−0.19	0.41 *	1						
**Act.Av.**	0.13	0.39 *	−0.01	−0.32	0.43 **	0.87 **	1					
**FoI**	0.36 *	0.16	0.30	0.17	0.22	0.54 **	0.16	1				
**SFMPQ**	0.13	0.31	0.005	−0.009	0.43 **	0.37 *	0.33 *	0.14	1			
**PPI**	0.25	0.36 *	0.06	−0.12	0.37 *	0.52 **	0.54 **	0.06	0.63 *	1		
**VAS**	0.15	0.17	−0.15	0.25	0.33	0.42 **	0.36	0.38	0.51 **	0.67 **	1	
**PD**	0.27	0.20	0.19	−0.04	0.30	0.51 **	0.52 **	0.22	0.25	0.25	-	1

PBPI, Pain Beliefs and Perceptions Inventory; TC, Time Constancy; A, Acceptance; S, Self-blame; M, Mystery; TSK, Tampa Scale for Kinesiophobia; Act.Av., Activity Avoidance; FoI, Fear of Injury; SFMPQ, Short-Form McGill Pain Questionnaire; PPI, Present Pain Intensity; VAS, Visual Analogue Scale; PD, Pain Duration; * *p* < 0.05, ** *p* < 0.01.

## Data Availability

Dataset available on request from the authors.

## Abbreviations

The following abbreviations are used in this manuscript:

PBPI	Pain Beliefs and Perceptions Inventory
TSK	Tampa Scale for Kinesiophobia
SF-MPQ	Short-Form McGill Pain Questionnaire
PPI	Present Pain Intensity
VAS	Visual Analogue Scale
TC	Time Constancy
A	Acceptance
S	Self-blame
M	Mystery
Act.Av.	Activity Avoidance
FoI	Fear of Injury
PD	Pain Duration
